# Loss of Auditory Activity Modifies the Location of Potassium Channel KCNQ5 in Auditory Brainstem Neurons

**DOI:** 10.1002/jnr.23516

**Published:** 2014-11-24

**Authors:** Elena Caminos, Elisabet Garcia-Pino, Jose M Juiz

**Affiliations:** 1 Instituto de Investigación en Discapacidades Neurológicas (IDINE), Facultad de Medicina, Universidad de Castilla-La ManchaAlbacete, Spain; 2 Institute of Biology, Freie Universität BerlinBerlin, Germany

**Keywords:** auditory system, cochlear ablation, Kv7.5, rat, TTX

## Abstract

KCNQ5/Kv7.5, a low-threshold noninactivating voltage-gated potassium channel, is preferentially targeted to excitatory endings of auditory neurons in the adult rat brainstem. Endbulds of Held from auditory nerve axons on the bushy cells of the ventral cochlear nucleus (VCN) and calyces of Held around the principal neurons in the medial nucleus of the trapezoid body (MNTB) are rich in KCNQ5 immunoreactivity. We have previously shown that this synaptic distribution occurs at about the time of hearing onset. The current study tests whether this localization in excitatory endings depends on the peripheral activity carried by the auditory nerve. Auditory nerve activity was abolished by cochlear removal or intracochlear injection of tetrodotoxin (TTX). Presence of KCNQ5 was analyzed by immunocytochemistry, Western blotting, and quantitative reverse transcription polymerase chain reaction. After cochlear removal, KCNQ5 immunoreactivity was virtually undetectable at its usual location in endbulbs and calyces of Held in the anteroventral CN and in the MNTB, respectively, although it was found in cell bodies in the VCN. The results were comparable after intracochlear TTX injection, which drastically reduced KCNQ5 immunostaining in MNTB calyces and increased immunolabeling in VCN cell bodies. Endbulbs of Held in the VCN also showed diminished KCNQ5 labeling after intracochlear TTX injection. These results show that peripheral activity from auditory nerve afferents is necessary to maintain the subcellular distribution of KCNQ5 in synaptic endings of the auditory brainstem. This may contribute to adaptations in the excitability and neurotransmitter release properties of these presynaptic endings under altered input conditions. © 2014 The Authors. Journal of Neuroscience Research Published by Wiley Periodicals, Inc.

Neurons in central auditory nuclei provide a useful model for studying neuronal adaptations to input deprivation. Removal of peripheral input from the cochlea and cochlear nerve has well-documented effects on the survival, structure, and function of neurons in the auditory brainstem (Born and Rubel, 1985; Hashisaki and Rubel, 1989; Moore, 1990; Tierney and Moore, 1997; Tierney et al., 1997; Mostafapour et al., 2000; Hildebrandt et al., 2011; Fuentes-Santamaría et al., 2013). The most dramatic effect is seen with cochlear removal before hearing onset, which results in significant loss of second-order auditory neurons in the cochlear nucleus (CN; Rubel et al., 2004). However, neurons in the mature auditory brainstem appear to survive deprivation of synaptic signals from the auditory nerve, but their “survival status,” i.e., molecular and cellular adaptations to absence of peripheral auditory activity, is still difficult to understand. Some potential reparative mechanisms occur following cochlear removal in the CN (Fuentes-Santamaría et al., 2007,2013). Studies that sought to test the influence of peripheral auditory input on the structure and function of postsynaptic neurons have focused on the cellular or molecular mechanisms common to most central neurons, such as apoptosis gene regulation, changes in RNA and protein synthesis, or regulation of cytoskeletal proteins (Born and Rubel, 1988; Pasic and Rubel, 1989; Sie and Rubel, 1992; Rubel et al., 2004; Harris and Rubel, 2006; Lu et al., 2007; Harris et al., 2005,2008).

The present study helps in understanding adaptive mechanisms of neuronal excitability and firing to changes in activity imposed by diminished or absent excitatory inputs. The KCNQ (or Kv7) family of voltage-dependent potassium channels may be important for signal processing by auditory neurons (Kharkovets et al., 2000; Navarro-López et al., 2009; Huang and Trussell, 2011). Mutations in KCNQ4 channels have been linked to deafness (Biervert et al., 1998; Kubisch et al., 1999; Kharkovets et al., 2000), but specific roles for KCNQ5 are still unknown. There is increasing evidence that the gene or protein expression of potassium channels is affected by early interruption of peripheral input to the auditory brainstem around the time of hearing onset (Lu et al., 2004; Caminos et al., 2005; Garcia-Pino et al., 2010). Previously, we showed that KCNQ5 is located in both the endbulbs of Held in the anteroventral CN (AVCN) and the calyces of Held in the medial nucleus of the trapezoid body (MNTB; Caminos et al., 2007). Both are involved in transmitting acoustic timing information. Localization of KCNQ5 in these excitatory endings suggests the regulation of resting properties, excitability, and transmitter release adapted to preserve timing faithfully. Roles in controlling resting properties and neurotransmitter release at the calyx of Held have been reported for KCNQ5 (Huang and Trussell, 2011). Determining whether the integrity of afferent inputs affects neuronal expression, distribution, and function of such specialized membrane molecules is of much interest.

For these purposes, the current study investigates whether the localization pattern of KCNQ5 in the giant synaptic terminals is altered when the afferent input to the parent neurons is lost in a posthearing mature animal, which would suggest regulation by ongoing activity. As described for developing auditory neurons, a mature-like localization pattern of KCNQ5 at the giant excitatory endings requires normal auditory peripheral activity onset (Garcia-Pino et al., 2010). Our results support the conclusion that neuronal activity is required to maintain the subcellular distribution of this potassium channel in the mature auditory brainstem.

## Materials and Methods

### Experimental Animals

Seventy-nine Wistar rats of both genders (Charles River Laboratories, Barcelona, Spain), with ages ranging from postnatal day (P) 30 to P70, were used in the present work. All procedures involving use of animals were supervised by the Animal House Facility of the University of Castilla-La Mancha and were approved by the Ethics Committee for Experimental Animal Welfare from the University of Castilla-La Mancha. These studies were conducted in accordance with European and Spanish laws (Directive 2010/63/UE and Real Decreto 53/2013, respectively).

### Cochlear Ablation

Bilateral cochlear removal was performed on P30 rats. Animals were deeply anesthetized with a mixture of ketamine (100 mg/kg; Parke-Davis, Alcobendas, Spain) and 2% xylazine (10 mg/kg; Dibapa, Barcelona, Spain). Normal body temperature was maintained during the surgical procedure with a heating pad. A retroauricular incision was made in the skin under aseptic conditions, and the bulla was opened until the cochlea was visible. The bony cochlea wall was perforated and its contents were removed, including the modiolus containing the spiral ganglion. After surgery, the skin was sutured and animals were allowed to recover until they regained consciousness. They were then housed under the same conditions as the matched control animals. After postoperative survival periods of 3, 10, or 40 days, unmanipulated controls (n = 8), sham controls (animals that received retroauricular incisions but did not undergo cochlear destruction; n = 3), and ablated rats (n = 12) were used for immunocytochemistry and histochemistry (Fluoro-Jade) as described below. In addition, 24 rats (12 experimental and 12 controls) were used for Western blotting, and 24 rats (12 experimental and 12 controls) were used for quantitative real-time polymerase chain reaction (PCR), as detailed in the corresponding sections. The extent of cochlear ablation was assessed by a macroscopic inspection of the dissected bulla, and only those cases with no visible cochlear remnants were included in the study. Anticalretinin immunocytochemistry and Fluoro-Jade staining (see below) were also carried out to check auditory nerve degeneration. Auditory brainstem response (ABR) recordings were performed to test hearing thresholds following bilateral cochlear ablation.

### Tetrodotoxin Application

Electrical activity in the auditory nerve was unilaterally blocked by using tetrodotoxin (TTX; 0.1 mM diluted in distilled water; Tocris Bioscience, Bristol, United Kingdom). On P30, four rats were deeply anesthetized with ketamine and xylazine (see above). Normal body temperature was maintained throughout the procedure. Next, 0.5–1 µl TTX was injected, through a hole that had been drilled into the left bulla, with a sterile syringe with an integrated needle of 0.33 × 12 mm (Braun, Frankfurt, Germany).

As controls, two animals were unilaterally injected with 0.5–1 µl distilled water into the bulla. Two hours after surgery, TTX-treated rats and vehicle-injected controls were sacrificed, and their brains were processed for immunocytochemistry. In addition, two rats were used as unmanipulated controls. ABR was measured immediately before and 1 hr after the TTX and distilled water injections (see below).

### ABR

ABR recordings were performed as previously described (Zheng et al., 1999; Cediel et al., 2006). Rats were anesthetized with ketamine (100 mg/kg) and xylazine (10 mg/kg) and placed in a sound-attenuating, electrically shielded booth (EYMASA/INCOTRON S.L., Barcelona, Spain) that was inside a sound-attenuating room. Animals were areflexive during the whole procedure. Normal temperature was recorded with a rectal probe and maintained at 37.5°C ± 1°C with a nonelectrical heating pad. Stainless-steel needle recording electrodes were placed subdermally at the vertex and ventrolaterally to the left and right ears. Click or tone stimuli were digitally generated in SigGeRPTM software (Tucker-Davis Technologies, Tulsa, OK) and calibrated in SigCal software with an ACO Pacifics one-fourth-inch microphone (Tucker-Davis Technologies). The duration of the click or tone burst stimuli was 0.1 msec, and three different frequencies were applied (8, 16, and 32 kHz). The response was analyzed in BioSigRPTM software (Tucker-Davis Technologies). To determine the auditory threshold level, evoked responses were recorded at the 10–90-dB sound pressure level (SPL) in 10-dB steps from the maximum intensity of 90 dB SPL. The auditory threshold was defined as the stimulus intensity that evoked waveforms with peak-to-peak voltage 2 standard deviations above background activity (measured before stimulus onset).

### Immunocytochemistry

For tissue processing, rats were deeply anesthetized with ketamine (100 mg/kg) and xylazine (10 mg/kg) and transcardially perfused with 0.9% saline and 2% paraformaldehyde in 0.1 M phosphate buffer (PB), pH 7.3. Brains were dissected and postfixed for 2 hr at 4°C in the same fixative, washed in PB, transferred into PB containing 30% sucrose, and embedded in Tissue Tek (Leica, Wetzlar, Germany). Serial coronal sections were cut at 16 µm with a cryostat (Leica) and mounted onto Superfrost slides (Kindler, Freiburg, Germany). Mounted sections were used for immunoperoxidase or Fluoro-Jade histochemistry procedures.

For peroxidase immunocytochemistry, sections were washed in phosphate-buffered saline, pH 7.3, containing 0.25% Triton X-100 (PBST) and immersed in 3% hydrogen peroxide in absolute methanol (10 min) to block endogenous peroxidase activity. After having been washed in PBST, sections were preincubated for 1 hr at room temperature (RT) with blocking solution containing PBST and 1% bovine serum albumin (BSA; fraction V; Sigma-Aldrich, Steinheim, Germany). Sections were then incubated with either mouse anticalretinin monoclonal (1:500) or rabbit anti-KCNQ5 polyclonal (1:1,000) primary antibodies, diluted in PBST-BSA, for 15 hr at RT in a humidified chamber. A list of primary antibodies with their immunological features, commercial sources and dilutions can be found in TableI. After incubation with the primary antibodies, tissues were washed with PBST and incubated for 1 hr with the corresponding secondary antibodies, biotinylated anti-mouse IgG for calretinin or biotinylated anti-rabbit IgG for KCNQ5 (Vector, Burlingame, CA), diluted at 1:200 in PBST-BSA. Sections were then washed in PBST and incubated with the avidin–biotin–peroxidase complex (ABC Kit; Vector) diluted at 1:250 in PBST-BSA. Sections were washed once with PBS and twice with 0.1 M Tris-HCl and were subsequently immersed in 0.025% diaminobenzidine and 0.015% H_2_O_2_ in 0.1 M Tris-HCl to reveal peroxidase activity. Finally, sections were dehydrated and mounted with Entellan (Merck, Darmstadt, Germany). Sections were examined and images were obtained with a Nikon (Tokyo, Japan) Eclipse 80i microscope equipped with a DXM 1200 C digital camera in ACT-1 image acquisition software (Nikon). The original images were digitally processed for composing images in figures with Adobe Photoshop CS3 (Adobe Systems, San Jose, CA). Three sets of controls were performed to test detection specificity: 1) omission of primary antisera by replacement with PBS-BSA, 2) omission of secondary antibodies, and 3) omission of the ABC reagent. None of these controls gave specific labeling. Control and ablated tissue or control tissue and TTX treatment were processed together in each experimental procedure.

**Table I:** Primary Antibodies Used for Immunocytochemistry and Western Blotting

Antigen	Immunogen (species/sequence)	Host species/mono-polyclonal	Manufacturer/catalog No./clone	Dilution^a^
KCNQ5 (or Kv7.5)	Fusion proteins from transformed E. coli strains/aa M1–R88	Rabbit/polyclonal	Millipore, Temecula, CA/AB5599	IR: 1:1,000 WB: 1:1,000
α-Calretinin	Recombinant human calretinin-22k/aa 1–178	Mouse/monoclonal	Swant, Bellizona, Switzerland/6B3/clone 6B3	IR: 1:500 WB: 1:1,000
GAPDH	Purified rabbit muscle GAPDH/whole molecule	Mouse/monoclonal	Applied Biosystems, Foster City, CA/AM4300/clone 6C5	WB: 1:2,000

a IR, immunocytochemistry; WB, Western blotting.

To analyze the effects of TTX application, semiquantitative image analysis was employed for the immunolabeled coronal sections of the MNTB from four animals injected unilaterally with TTX (two vehicle-injected controls and two unmanipulated rats). Between six and eight caudorostral digital photographs of the MNTB were taken with the Nikon Eclipse 80i microscope equipped with the DXM 1200 C digital camera in the Nikon ACT-1 capture software. All images were captured at the same magnification (40 × 10, N.A. 0.75). The illumination conditions of the microscope and the setting of the digital camera software were unchanged throughout the procedure. Images were analyzed in Adobe Photoshop CS3 and ImageJ (NIH, Bethesda, MD; http://rsb.info.nih.gov/ij/). Size was calibrated, and a grid with square fields of 75 × 75 µm was overlaid on each image. Six fields were randomly selected, which were always the same for all the MNTB images. In each field, all the visible cell body profiles were marked and counted. Immunostained regions of interest, i.e., punctate or synaptic ending-like labeling around cell bodies, were segmented by conventional thresholding methods (a defined threshold was kept constant across all the tissue sections). Segmentation resulted in the generation of eight-bit images, which were used to count the number of immunostained pixels automatically around each cell body profile. Data were expressed as the average number of immunostained pixels/average of number of cell body profiles. The ipsilateral and contralateral sides to the unilateral injections were compared. Differences between the means of the TTX-treated and the control groups were analyzed by one-way ANOVA, and significant differences between the experimental group and the control groups were detected by a Scheffé post hoc test.

### Fluoro-Jade Histochemistry

Fluoro-Jade histochemistry was performed to analyze neuronal degeneration in brainstem sections after cochlear ablation (Schmued et al., 1997; Schmued and Hopkins, 2000). Fluoro-Jade B (Chemicon, Temecula, CA) was used for this purpose, with excitation and emission peaks in distilled water of 480 nm and 525 nm, respectively.

Brain sections from ablated and control animals were mounted onto Superfrost slides and were fully air dried for 20 min. Afterward, the tissue was immersed in 100% ethanol for 3 min, followed by 1 min in 70% ethanol and 1 min in distilled water. Slides were then transferred to a solution of 0.06% potassium permanganate for 15 min, rinsed for 1 min with distilled water, and incubated with 0.001% Fluoro-Jade staining solution for 30 min in the dark. Degeneration was detected in Fluoro-Jade-stained sections under a Leica (Wetzlar, Germany) TCS-SP2 laser scanning confocal microscope.

### Western Blotting

Western blotting was carried out to determine the relative changes in the KCNQ5 and calretinin (used as the positive control) levels in the CN 40 days after cochlear ablation. Three control samples and three experimental samples were used. Each sample was pooled from four animals, which made a total of 12 deafened rats and 12 controls. The control and ablated tissues were processed together in each experimental procedure.

Rats were anesthetized with carbon dioxide in an appropriate chamber prior to decapitation. The brain was removed and CN were dissected out, frozen, and stored at −80°C. They were homogenized in 2 ml lysis buffer (250 mM sucrose, 10 mM Tris, 10 mM HEPES, and 10 mM EDTA, pH 7.2) containing a cocktail of protease inhibitors (Sigma-Aldrich). The homogenate was centrifuged at 10,000*g* for 10 min at 4°C, and the supernatant was ultracentrifuged at 68,600*g* for 1 hr at 4°C. The pellet, enriched in membrane proteins, was resuspended in 200 μl of the aforementioned buffer, aliquoted, and stored at −80°C. The protein concentration of the samples was determined with a Micro-BCA protein kit (Pierce, Rockford, IL). Twenty micrograms of whole-protein extract was electrophoresed on 8% sodium dodecyl sulfate polyacrylamide gels by using the Mini-Protean III system (Bio-Rad, Hercules, CA) for 90 min at 120 V. Gels were subsequently transferred onto nitrocellulose membranes (GE Healthcare Bio-Sciences, Uppsala, Sweden) for 1 hr at 20 V with a semidry blotter (Bio-Rad). Immunodetection was performed by first blocking nonspecific binding in the blots with TBST/milk (50 mM Tris, pH 7.5, 200 mM NaCl, 0.1% Tween 20, and 5% nonfat dry milk) for 1 hr at RT and incubation in the aforementioned buffer overnight at 4°C with the same primary antibodies as used for the immunocytochemistry procedure (1:1,000 rabbit anti-KCNQ5; 1:1,000 mouse anticalretinin). On the following day, blots were incubated with the corresponding secondary antibodies (1:1,000 horseradish peroxidase-conjugated goat anti-rabbit IgG or goat anti-mouse IgG; Pierce) for 1.5 hr at RT. Bound secondary antibodies were detected with an enhanced chemiluminescence assay (SuperSignal West dura substrate; Pierce) and scanned into a computer equipped with the LAS-Mini 3000 system (Fujifilm, Tokyo, Japan). Then, blots were incubated in Western blotting stripping buffer (Pierce) at 37°C for 30 min and exposed to antiglyceraldehyde-3-phosphate dehydrogenase (GAPDH; 1:2,000; Applied Biosystems, Foster City, CA; TableI) by using the same protocol described above. Densitometry for protein band quantification was performed in Quantity One 4.1 analysis software (Bio-Rad, Hercules, CA). Values were expressed as the ratio of the KCNQ5-band average gray levels to the average gray levels of the GAPDH bands. Data were obtained from three different samples in triplicate. Statistical significance was assessed by an unpaired *t*-test with Welch's correction, and *P* < 0.05 was considered statistically significant.

### Quantitative Reverse Transcriptase-Polymerase Chain Reaction

Quantitative reverse transcriptase-polymerase chain reaction (qRT-PCR) was carried out to test changes in the KCNQ5 mRNA levels in the CN after cochlear ablation. The RNA used in this study was obtained from 24 rats. Three different pools were collected for each age and condition, rats after cochlear ablation, P30 plus 3 hr after ablation, and P30 plus 40 days after ablation, with corresponding P30 and P70 controls. Each RNA pool contained the CN of two randomly assigned animals.

Rats were deeply anesthetized and decapitated. Brains were extracted, and, after removal of the overlying cerebellar flocculus, the entire CN was dissected out bilaterally. The tissue was immediately frozen in liquid nitrogen and stored at −80°C. Total RNA was isolated with Trizol (Invitrogen, Paisley, United Kingdom). Isolated RNA concentration and integrity were checked and further treated with RNase-free DNase (Promega, Madison, WI) to remove any genomic DNA. DNase-treated RNA was reverse transcribed by using Taqman reverse transcription reagents (Applied Biosystems).

Real-time PCR was carried out on an AB Prism 7000 System (Applied Biosystems). cDNA (1 μl of reverse transcription product for KCNQ5 and 1 μl of 1:100 reverse transcription product for 18S rRNA) was amplified by using Sybr green premix Ex Taq (perfect real time) reagents (Takara Bio, Otsu, Japan) in the presence of primer oligonucleotides specific for KCNQ5 and 18S rRNA. The PCR conditions were 95°C for 10 min, followed by 40 cycles consisting of 95°C for 15 sec and 60°C for 1 min. Quantification was performed by the comparative cycle threshold method (Livak and Schmittgen, 2001) with the 18S rRNA expression level as the internal control. Melting curves were also analyzed to ensure that only one product was obtained per primer pair. Primer sequences were designed in Primer Express software 2.0 (Applied Biosystems). Ablated and control rat tissues were processed together in each experimental procedure. Comparison between experimental and control groups (P30 vs. P30 plus 3 hr after ablation and P70 vs. P30 plus 40 days after ablation) was performed via Student's *t*-test, and *P* < 0.05 was considered statistically significant.

## Results

### ABR After Bilateral Cochlear Ablation

**Figure 1:**
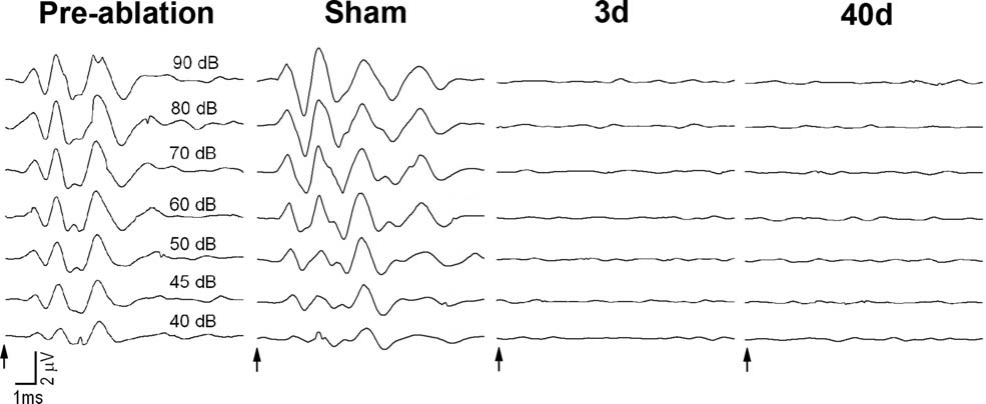
Representative ABR traces of an adult rat collected before and after cochlear removal. Before cochlear removal, five peak waves in response to click stimuli were recorded in rats at P30. No evoked responses were observed in rats on days 3 (3d) and 40 (40d) after cochlea removal. The sham control did not eliminate the ABR. Sound intensity is indicated (dB SPL). Arrows indicate the start of stimulation.

ABRs were measured to test whether the bilateral cochlear ablation procedure blocked central auditory activity. Both presurgery and unmanipulated control rats showed that the ABR recordings were within the normal values for Wistar rats, with an average ABR threshold to the click stimuli of 46.5 ± 4.9 dB SPL. Equally, sham surgery had no effect on ABR thresholds or latencies (Fig. 1). After cochlear removal, rats showed a complete elimination of any recordable ABR compared with the same response recorded before cochlear ablation (Fig. 1). These findings indicate that there was no generation of activity driven by auditory nerve input and that brainstem auditory nuclei did not generate activity of this kind after bilateral cochlear ablation at any survival time.

### KCNQ5 in the AVCN and MNTB After Bilateral Cochlear Removal

**Figure 2:**
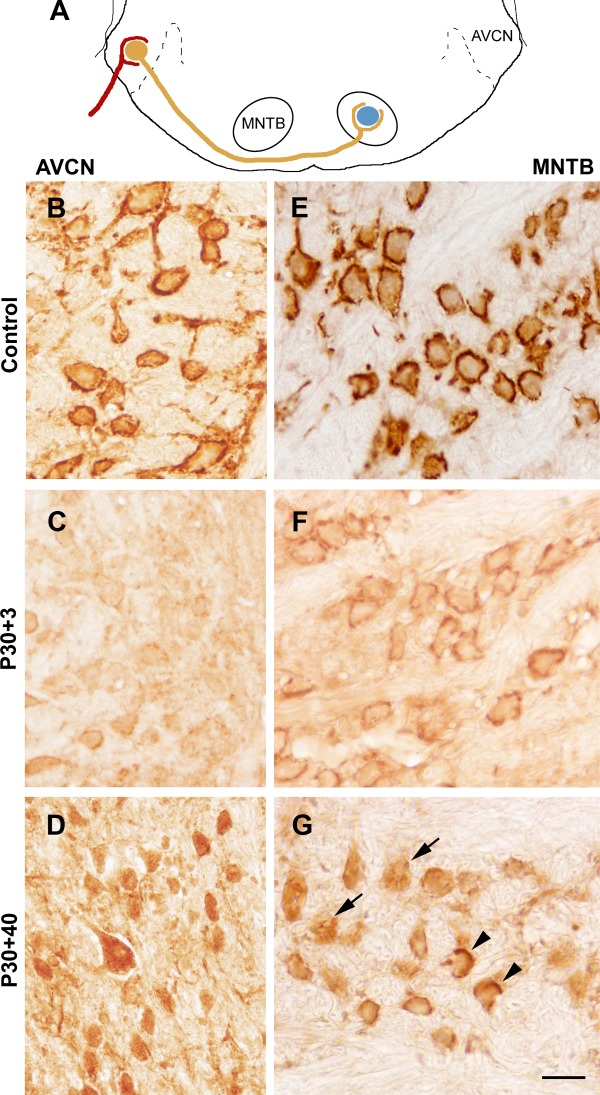
Effects of bilateral cochlear ablation on KCNQ5 distribution and expression in the AVCN and MNTB. A: Representative drawing with a simplified AVCN–MNTB circuit. B–D: Micrographs of coronal sections of the AVCN from a normal control and a rat after cochlear ablation. Control section shows immunolabeling in the synaptic endings around the cells bodies corresponding to endbulbs of Held (B). KCNQ5 immunoreactivity virtually had disappeared from the endings around AVCN neurons 3 days after cochlear removal (C). AVCN neurons intensely labeled 40 days after ablation (D). E–G: Micrographs illustrating KCNQ5 immunolabeling in the coronal sections of the MNTB in normal control rats and rats after cochlear removal. Representative section of a control animal shows immunolabeling in the synaptic terminals around the principal neurons corresponding to calyces of Held (E). Immunoreactive profiles detected 3 days after cochlea removal (F). KCNQ5 immunoreactive synaptic endings tended to disappear 40 days after ablation (G; arrows). Some cells show immunoreactivity in the endings around cell bodies (arrowheads). Scale bar = 20 µm. [Color figure can be viewed in the online issue, which is available at http://wileyonlinelibrary.com.]

Coronal sections at the AVCN and MNTB levels (Fig. 2A) were analyzed. In the AVCN of control animals, there were numerous bouton-like structures corresponding to the synaptic endings labeled for KCNQ5 (Fig. 2B). The AVCN sections from animals in which the cochlea had been removed at P30 displayed a loss of immunoreactive endings around cell bodies starting on day 3 and continuing to day10 after surgery, probably as a result of axonal degeneration. In these animals, a small number of cells showed a few KCNQ5-stained bouton-like structures around their somata (Fig. 2C). On day 40 following cochlear ablation, the KCNQ5-immunostained endings around AVCN neurons had almost completely disappeared, whereas immunoreactive cell bodies, previously not visible, were seen in the AVCN (Fig. 2D).

In the MNTB, KCNQ5 immunostaining in the control animals was concentrated in the synaptic terminals around cell bodies, with virtually no labeling in cell bodies or the neuropil (Fig. 2E). At 3 and 10 days after cochlear removal, immunoreactive endings were still seen around MNTB neurons (Fig. 2F), whereas very few isolated neurons showed immunoreactivity around their somata on day 40 after cochlear removal (Fig. 2G). At this same postcochlea removal time, and coinciding with the disappearance or attenuation of KCNQ5 immunoreactivity from presumptive calyces of Held, many cell bodies in the MNTB showed immunoreactivity for KCNQ5 that had not previously been detectable (Fig. 2G).

**Figure 3:**
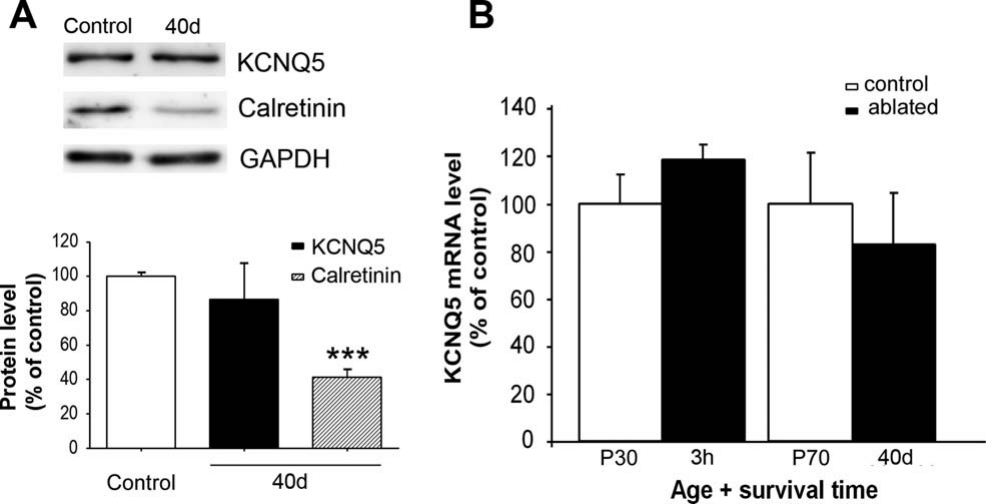
Analysis of the KCNQ5 protein and mRNA levels in the CN after cochlear ablation. A: Western blots show levels of KCNQ5 and calretinin in the CN extracts from unmanipulated controls and rats 40 days after bilateral cochlear ablation (40d). Detection of GAPDH was included as a loading control. No significant differences were found in KCNQ5 protein density 40 days after cochlear removal compared with the controls (P70). Calretinin expression levels were significantly reduced. Values are expressed as the relative fold change percentage compared with the controls. B: KCNQ5 mRNA expression levels in the CN of the unmanipulated control rats and the rats in which the cochleae had been removed. Quantitative real-time RT-PCR showed no significant differences in the relative levels of KCNQ5 mRNA between control rats and rats 3 hr (3h) and 40 days (40d) after cochlear ablation (*P* > 0.05). Bars represent mean ± SEM of nine independent determinations. ***P < 0.001.

Regulation of KCNQ5 levels 40 days after cochlear removal was also analyzed by Western blotting (Fig. 3A). The densitometry analysis revealed that the overall KCNQ5 protein density in the CN 40 days after cochlear removal was statistically similar to that of the controls (86.695% ± 25.15% compared with the control group; *P* = 0.5839, unpaired *t*-test with Welch's correction). Samples included tissue trace amounts from the dorsal cochlear nucleus (DCN). These results support the conclusion that de novo KCNQ5 immunolabeling in neuronal cell bodies of the AVCN 40 days after cochlear removal reflects protein accumulation at levels comparable to those found in normal animals in endbulbs of Held, which degenerated as a result of cochlear removal. As an internal positive control for Western blotting, we also tested the calretinin levels, which had dropped dramatically (about 60% compared with the control group; *P* < 0.001, unpaired *t*-test with Welch's correction) 40 days after bilateral cochlear ablation (Fig. 3A). This corroborated, at the same time, loss of the nerve fibers seen with immunocytochemistry and Fluoro-Jade labeling.

To test the regulation of the KCNQ5 mRNA expression levels in the CN after bilateral cochlear ablation, the KCNQ5 mRNA levels were measured by qRT-PCR 3 hr after ablation and on day 40 after ablation (Fig. 3B). No significant differences were observed in the KCNQ5 mRNA expression levels between the CN of the deafferented and the control rats.

DCN were included in the tissue samples. KCNQ5 expression was detected only in the punctate structures in the deep DCN layers and not in the cell bodies (Caminos et al., 2007).

### Time Course of Degeneration in the CN

**Figure 4:**
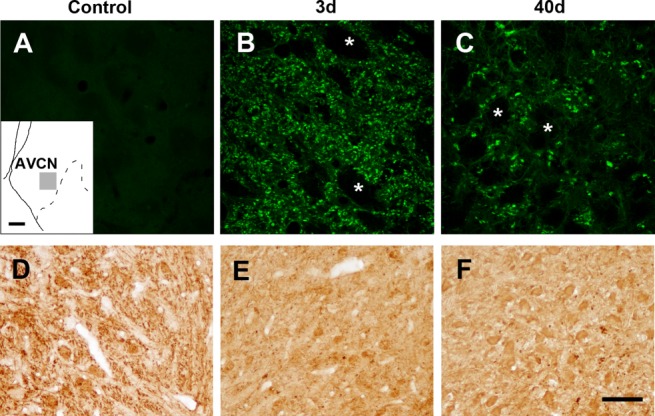
Time course of degeneration in the AVCN after bilateral cochlear ablation. Representative micrographs from coronal sections of the AVCN (see inset) from an unmanipulated control rat, a rat surviving 3 days after bilateral cochlear ablation, and a rat surviving 40 days after bilateral cochlear ablation. A–C: Micrographs illustrate Fluoro-Jade staining. Compared with the control (A), intense Fluoro-Jade staining is observed in the fibers of the AVCN from rats 3 (B) and 40 (C) days after ablation. Note that all the staining is concentrated in the fibers scattered in the neuropil and that the somata are not stained (asterisks). D–F: High magnifications illustrate the calretinin immunoreactivity pattern in the AVCN. Strong immunoreactivity is distributed in the fiber bundles in the neuropil of the control sections (D). Calretinin immunoreactivity in fibers greatly diminished at 3 and 40 days after cochlear removal (E,F, respectively). Scale bars = 100 µm in the inset; 20 µm for A–C; 50 µm in F (applies to D–F). [Color figure can be viewed in the online issue, which is available at http://wileyonlinelibrary.com.]

Fluoro-Jade staining helped determine the time course of fiber degeneration in the CN after bilateral cochlear ablation (Fig. 4A–C). Control sections of the AVCN showed only background fluorescence (Fig. 4A). On days 3, 10, and 40 after cochlear removal, the coronal sections of the CN exhibited degenerated terminal and fiber fields labeled with Fluoro-Jade, which occupied the nerve root and a large area of the CN (Fig. 4B,C). In contrast, cell bodies of CN neurons were not labeled with Fluoro-Jade at either stage (Fig. 4B,C), suggesting that no transneuronal degeneration took place, at least in this experimental time window.

Anticalretinin immunocytochemistry was performed simultaneously to test loss of primary auditory fibers after bilateral cochlear removal (Fig. 4D–F). The control animals showed immunoreactive bundles (better seen in the coronal sections of the cochlear root region) corresponding to auditory nerve fibers (Fig. 4D). In contrast, neither visible nor faint immunolabeling was observed at any time after cochlea removal (Fig. 4E,F).

Taken together, these findings support the conclusions that auditory nerve fibers degenerate quickly after cochlea removal and that the degeneration continues up to 40 days later. In contrast, cell bodies in the AVCN showed no signs of transneuronal degeneration.

### KCNQ5 Distribution in the AVCN and MNTB After the Intracochlear Application of TTX

**Figure 5:**
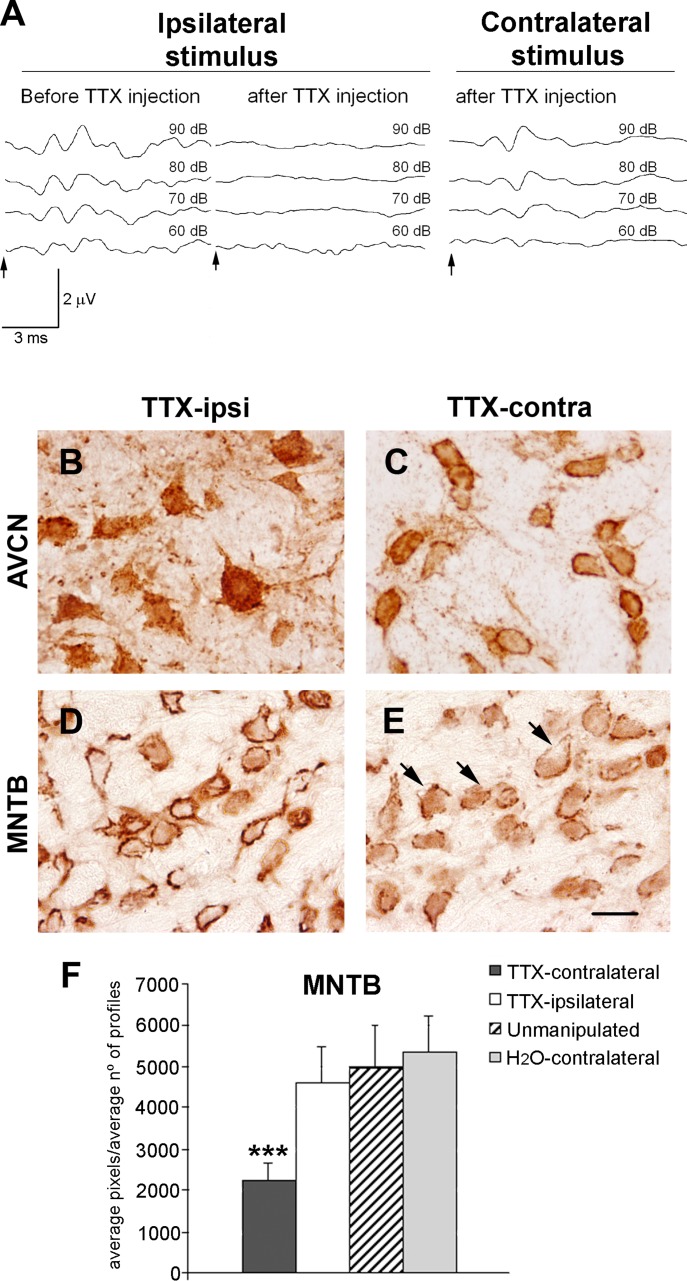
Effects of unilateral TTX injection into the cochlea on KCNQ5 immunoreactivity in the auditory brainstem. A: Representative ABRs to a 16-kHz tone-burst stimulus of an adult rat collected before and after TTX injection into the left bulla. ABRs to the 16-kHz tone stimulus delivered to the left ear were recorded from the left side before TTX injection. After TTX injection, ABRs were eliminated. After stimulation of the contralateral side, waves were present, though attenuated, and thresholds were higher. Sound intensity is indicated in dB SPL. Arrows indicate the start of the stimulus. B–E: Micrographs showing KCNQ5 immunoreactivity in the AVCN (B,C) and the MNTB (D,E) after the unilateral TTX injection. In the ipsilateral AVCN, immunoreactive synaptic endings disappeared around cell bodies, but cell bodies became intensely immunoreactive (B). In the contralateral AVCN of the TTX-treated animals, immunolabeling around the AVCN neurons is observed (C), as seen in the unmanipulated animals and in the vehicle-injected rats (distilled water). The MNTB on the ipsilateral side of the TTX injection shows the abundant immunoreactive synaptic endings surrounding MNTB neurons (D). The contralateral MNTB exhibits the areas where KCNQ5 immunoreactivity disappeared from endings (arrows; E). F: Representative histogram shows the number of immunostained pixels counted on coronal sections of the MNTB (mean ± SD). Note the significant difference between the contralateral side of one animal injected with TTX compared with its ipsilateral side, the unmanipulated animal, and the vehicle-treated control rats. ***P < 0.001. TTX-ipsi, ipsilateral side to TTX injection; TTX-contra, contralateral side to the TTX injection. Scale bar = 20 µm. [Color figure can be viewed in the online issue, which is available at http://wileyonlinelibrary.com.]

The unilateral TTX injections in the left cochlea resulted in the complete elimination of normal ABR waves after ipsilateral stimulation. Stimulation of the contralateral ear resulted in a slight but not statistically significant increase in the auditory thresholds as well as a modest attenuation of wave amplitudes, particularly in waves III and IV (Fig. 5A). These results support the conclusion that TTX application blocks central auditory responses to sound by blocking auditory nerve activity.

The effects of the acoustic activity blockade by TTX application on the KCNQ5 distribution are shown in representative photomicrographs of the coronal sections of the AVCN from an animal that survived 2 hr after TTX injection (Fig. 5B,C). After the unilateral TTX application, the ipsilateral AVCN showed loss of KCNQ5 immunolabeling in the endings around cell bodies, with KCNQ5 immunostaining appearing in cell bodies (Fig. 5B). In contrast, the contralateral AVCN of rats displayed the normal pattern of immunolabeling concentrated in the synaptic terminals around cell bodies (Fig. 5C). No differences in KCNQ5 distribution were observed between the vehicle (intracochlear injection of distilled water) and the unmanipulated control rats.

The TTX unilateral injections were expected to block peripherally driven activity in the contralateral MNTB significantly. Thus, comparing the contralateral and ipsilateral MNTB should provide another measure of the effects of a lack of (or limited) activity in the cochlear nucleus neurons, which give rise to the calyces of Held, on the distribution of KCNQ5 in these synaptic endings. In the MNTB (Fig. 5D,E) ipsilateral to the injection side, there were numerous immunoreactive endings around the neuronal cell bodies (Fig. 5D). This distribution was similar to that seen in the control animals (Fig. 2E). In contrast, the contralateral MNTB, which is the target of some of the AVCN projections originating in the neurons affected by intracochlear TTX injection, displayed a loss of KCNQ5 immunoreactivity in the synaptic endings surrounding cell bodies (Fig. 5E).

Differences in immunolabeling were confirmed by counting the number of labeled pixels in digital images from the ipsilateral and contralateral MNTB in image analysis software. As shown in Figure 5F, the differences in the average number of labeled pixels per average number of cell body profiles between the MNTB contralaterally and ipsilaterally to the TTX injection were analyzed by one-way ANOVA. Significant differences were detected between the experimental group injected with TTX and the control groups (contralateral 2,235.97 ± 440.75, ipsilateral 4,594.29 ± 877.24; *P* < 0.001). No statistically significant differences were found in the average number of labeled pixels among the ipsilateral and contralateral MNTB and the controls, the vehicle-injected animals (5,329.82 ± 886.62; *P* < 0.05), or both of the MNTBs of the unmanipulated animals (4,960.16 ± 1,045.63; *P* < 0.05).

## Discussion

This study provides evidence that, when the auditory system is mature, KCNQ5 localization in the auditory brainstem nuclei is susceptible to loss of auditory activity, even when the auditory nerve is structurally intact.

### Redistribution of KCNQ5 in Auditory Brainstem Neurons After Removing Peripheral Excitatory Input

ABRs confirmed functional loss of auditory nerve input after bilateral cochlear ablations in adult rats. KCNQ5 immunoreactivity in the synaptic endings of the AVCN disappeared 10 days later after cochlear removal. This result may be a direct consequence of terminal and fiber degeneration. A considerable decline in the excitatory synaptic contact zones in VCN neurons 7 days after unilateral cochlea removal has been shown in adult rats (Hildebrandt et al., 2011). More relevantly, numerous cell bodies in the AVCN were intensely immunolabeled for KCNQ5 on day 40 after cochlear ablation, which were not seen in the control animals. An adequate interpretation of this finding requires examining the MNTB.

The MNTB in the superior olivary complex is a main recipient of axonal projections from the globular/bushy cells in VCN, which give rise to calyces of Held on principal MNTB neurons (Cant and Benson, 2003). Like their endbulb counterparts in the AVCN, calyces of Held are enriched in KCNQ5 immunoreactivity (Caminos et al., 2007; Garcia-Pino et al., 2010). This predicts the participation of KCNQ5 in excitability regulation at these synaptic endings (Huang and Trussell, 2011). Localization of KCNQ5 at the calyx of Held was progressively lost after cochlear ablation. Indeed, 40 days after cochlear removal there was little or no labeling for KCNQ5 in calyces of Held. Unlike the endbulbs in the AVCN, this is not attributable to primary degeneration because calyces, or their parent cell bodies in the VCN, were not directly affected by cochlear removal. In fact, no evidence of degeneration was found with Fluoro-Jade in the cell bodies of the VCN. Cochlear removal is not expected to have a major effect on the survival of central neurons in adult animals because, after the critical period that coincides with hearing onset, neurons in the CN no longer show such strong dependence on their excitatory afferents to survive (Hashisaki and Rubel, 1989; Moore, 1990; Tierney and Moore, 1997; Harris and Rubel, 2006). Actually, the structure of the MNTB and the size of neuronal cell bodies in the contralateral MNTB were preserved 24 hr after cochlear ablation or eighth nerve activity blockade by TTX (Pasic et al., 1994).

Therefore, inasmuch as there is no evidence that the inputs to the MNTB degenerate, loss of KCNQ5 immunolabeling from calyces of Held must be attributable primarily to absence of afferent excitatory activity on their neurons of origin in the CN. The fact that, in parallel with KCNQ5 disappearing from calyces of Held, a large number of cell bodies in the AVCN showed KCNQ5 immunoreactivity (which were not present under normal hearing conditions) must be seen in the possible activity-dependent redistribution context of this potassium channel. These cell bodies, immunolabeled de novo for KCNQ5, resemble the distribution of this potassium channel, as seen in developing animals before airborne sound activates brainstem auditory neurons upon hearing onset (Garcia-Pino et al., 2010). Increased KCNQ5 immunoreactivity was also seen in MNTB cell bodies after cochlear ablation or TTX injection. Although this finding was not further analyzed, it suggests that the activity-dependent redistribution of KCNQ5 affects neurons at different auditory brainstem levels.

One possible explanation for loss of KCNQ5 from synaptic endings, with concurrent accumulation of protein in cell bodies in the AVCN, is that absence of peripheral activity may affect KCNQ5 protein turnover at the calyx of Held and lower its levels through increased protein degradation, altered targeting, altered rates of selective axonal transport to the ending, diminished protein synthesis, or any combination of these. Activity-dependent protein degradation at central synapses has been reported, and the central role of regulated proteolysis through the ubiquitin–proteasome complex has been highlighted (Segref and Hoppe, 2009; Jiang et al., 2010). It is also well known that ongoing activity of afferent inputs, or its absence, dynamically regulates the synaptic structure and function by directing the targeting and removal of proteins at the synapse, at least in young animals (Rubel et al., 2004). It is not known, however, whether lack of activity selectively regulates the synaptic targeting and transport of potassium channels (Gu and Barry, 2011). Diminished KCNQ5 immunolabeling in calyces as early as 2 hr after TTX injection indicates that the removal of this potassium channel from synaptic endings by activity blockade quickly takes place. Such a fast effect suggests that, perhaps, lack of activity directly interferes with the KCNQ5 turnover at the synaptic ending by increasing protein degradation rates. This, however, does not exclude other mechanisms, as suggested by the presence and distribution of KCNQ5 in the AVCN before and after removing peripheral input.

In short, the combined results of immunocytochemistry and Western blotting suggest a compensatory increase in KCNQ5 protein synthesis and/or processing at the cell body, be it successful or not. Such a hypothetical increase in KCNQ5 synthesis does not require increased mRNA levels, as shown by qRT-PCR, which suggests that lack of activity may trigger posttranscriptional regulation. Alternatively, KCNQ5 might accumulate in neuronal cell bodies because the transport rates to and from the ending might be affected by lack of afferent activity. Any of these possibilities, either alone or in combination, may result in KCNQ5 accumulation in neuronal cell bodies.

DCN were included in tissue samples for Western blotting and RT-PCR. KCNQ5 expression was detected only in punctate structures in the deep DCN layers and not in cells bodies (Caminos et al., 2007). Analysis of KCNQ5 immunoreactivity in DCN was not incorporated into the current work because it is beyond the main question about activity-dependent changes in KCNQ5 distribution resulting from DCN neurons that do not project to MNTB.

Further experiments must be conducted to address the cellular mechanisms involved in the activity regulation of KCNQ5 levels and distribution. Whatever these mechanisms are, they likely form part of a specific adaptive response and are not the consequence of neuronal damage, or they may form part of a generic neuronal reaction to diminished activity, given that other synaptic proteins such as synaptophysin display different behavior in the CN after removal of afferent inputs (Fuentes-Santamaría et al., 2007).

### Activity Is Essential To Regulate the Subcellular Distribution of KCNQ5 in Auditory Neurons of the Brainstem

The fact that afferent activity from the periphery is a primary factor in regulating the levels and subcellular distribution of KCNQ5 in neurons of the auditory brainstem was shown by blocking auditory nerve activity with unilateral TTX injections into the cochlea. These TTX studies were very short term because it has been documented that TTX can produce changes in the central auditory pathway as early as 30 min (in avian species) or 1 hr (in gerbils; Pasic and Rubel, 1989; Sie and Rubel, 1992). The blocking effects of TTX on action potential generation and spread are not accompanied by damage to axons or synaptic endings (Pasic and Rubel, 1989). To determine whether activity is directly responsible for changes in the KCNQ5 distribution, we analyzed the contralateral MNTB, which receives direct input from the VCN of the injected side. KCNQ5 immunoreactivity tended to disappear from the endings in the contralateral MNTB but not in the ipsilateral MNTB. KCNQ5 immunostaining patterns were the same in the MNTB when deafferentation was performed by bilateral ablation. Taken together, these results reveal that the neuronal activity carried by auditory nerve afferents modulates the long-term deployment of KCNQ5 in excitatory terminals.

Altered glutamate neurotransmission itself, or other factors co-released with glutamate, may be involved in such regulation (Rubel et al., 2004). Although the expression of other voltage-gated channels, such as Kv1.1 and Kv3.1, has been shown to be regulated by activity in the CN (Lu et al., 2004), this is the first experimental evidence to suggest that the distribution of a potassium channel depends on ongoing neuronal activity. These activity-dependent changes in distribution may regulate the function in response to lack of or limited neuronal excitation.

### A Role for the Activity-Dependent Downregulation of KCNQ5 in Calyces of Held

The location of KCNQ5 immunoreactivity in calyces of Held predicts roles for this channel in excitability regulation. It has been reported that KCNQ5 actually regulates resting properties and glutamate release probability in calyces of Held (Huang and Trussell, 2011). Removal of KCNQ5 from the calyx, induced by diminished peripheral activity, likely shifts the resting membrane potential to less negative levels. When operating at resting potentials closer to the threshold, action potential spiking is facilitated, and glutamate release probability increases over time. This may be a way to adapt excitability in the presynaptic ending to diminished or absent excitatory inputs. When KCNQ5 is absent, the calyx membrane might be driven to threshold by weaker potential trains and may activate glutamate release. Therefore, diminished excitatory input to the presynaptic neuron should still be able to induce activation or firing in the postsynaptic neuron, although the trade-off may be timing degradation. This is an appealing hypothesis, but it requires further experimental validation.

## Conclusions

This study demonstrates that the synaptic localization of potassium channel KCNQ5 in mature rat auditory brainstem nuclei is susceptible to loss of auditory activity. KCNQ5 immunoreactivity virtually disappeared from its normal location in calyces of Held in the MNTB and appeared in cell bodies in the AVCN. These changes may form part of an adaptive neuronal response rather than being the consequence of neuronal damage. Thus, activity carried by auditory nerve afferents seems to modulate long-term KCNQ5 deployment in excitatory terminals. This may contribute to adaptation in the excitability and neurotransmitter release properties of these presynaptic endings in response to altered input situations.
